# Juvenile Psoriatic Arthritis (JPsA): juvenile arthritis with psoriasis?

**DOI:** 10.1186/1546-0096-11-11

**Published:** 2013-03-15

**Authors:** Yonatan Butbul Aviel, Pascal Tyrrell, Rayfel Schneider, Sandeep Dhillon, Brian M Feldman, Ronald Laxer, Rotraud K Saurenmann, Lynn Spiegel, Bonnie Cameron, Shirley ML Tse, Earl Silverman

**Affiliations:** 1 Division of Rheumatology, SickKids Hospital, Hospital for Sick Children Research Institute, University of Toronto, Toronto, ON, Canada; 2 The Hospital for Sick Children, Departments of Pediatrics and Immunology, Departments of Pediatrics, Medicine, Health Policy Management and Evaluation, Toronto, Canada; 3 Dalla Lana School of Public Health, University of Toronto, Toronto, ON, Canada

## Abstract

**Background:**

Following the introduction of the ILAR criteria for juvenile idiopathic arthritis, juvenile psoriatic arthritis (JPsA) has become a better recognized category within the inflammatory arthritides of childhood. There are fewer reports describing the characteristics and long-term outcome of patients with JPsA than other subtypes of JIA.

The aim of our study was to determine the long-term outcome and clinical course of patients with juvenile psoriatic arthritis (JPsA) and to define subgroups of JPsA.

**Methods:**

Clinical records of all patients meeting criteria for JPsA were reviewed and divided into 4 groups depending on their clinical features and onset type. Patient characteristics and clinical features at onset and during follow-up were determined.

**Results:**

The cohort consisted of 119 patients: 65 with oligoarticular-onset (55%; persistent 44 and extended 21), 34 (29%) with RF(-) and 4 (3%) RF(+) polyarticular and 16 (13%) enthesitis-related arthritis (ERA). At diagnosis patients with ERA were oldest and more commonly male (p=0.001 and =0.01 respectively). Patients with a polyarticular course had more involvement of small joints of the hands and wrist when compared to patients with persistent oligoarticular and ERA (p<0.001) while patients with ERA had more hip and sacroiliac arthritis (p<0.001 for both). Nail changes were seen in 66 patients (57%) and were associated with DIP involvement (p=0.0034).

Outcome: Time to first inactive disease on, but not off, therapy was significantly longer among patients with polyarticular course when compared to oligoarticular and ERA (p=0.016 and p=0.48 respectively). Patients with polyarticular course more frequently had contractures during follow-up than other groups (p=0.01).

**Conclusion:**

The long-term outcome of with JPsA was generally good. Patients with JPsA did not appear to form distinct sub-group of patients but rather resembled JIA patients with onset types without psoriasis.

## Background

Following the introduction of the ILAR criteria for juvenile idiopathic arthritis, juvenile psoriatic arthritis (JPsA) has become a better recognized category within the inflammatory arthritides of childhood. Patients defined as having JPsA, within the ILAR classification, must have a persistent arthritis of greater than 6 weeks with onset of the arthritis prior to age 16 and either the presence of a psoriatic rash, or in the absence of rash, at least 2 of the following minor criteria: first degree relative with psoriasis, nail pitting or onycholysis, and dactilytis [1]. Prior to the ILAR criteria another classification scheme was used: the Vancouver Criteria [2]. This classification scheme differs from the ILAR classification by the following additional minor criteria: a) the psoriasis could be in first-degree or second-degree relative and b) the presence of a psoriatic-like rash is sufficient for classification of JPsA. According to the Vancouver criteria definite JPsA is defined as arthritis with either psoriasis or at least 3 minor criteria; patients with only 2 minor criteria were defined as probable JPsA. This latter category is not present in the ILAR Classification.

There are fewer reports describing the characteristics and long-term outcome of patients with JPsA than other subtypes of JIA and no report solely using the ILAR Criteria. In addition, some were review articles, reviewed overlapping subsets of patients or were small cases series [2-13]. As more than one classification system for JPsA has been used, it can be confusing to compare different studies and, as recently as 2008, no single classification was agreed upon or universally accepted [12].

The aims of this study were to determine the long-term joint outcome and functional status of children with JPsA and to define subgroups of JPsA.

## Patients and methods

### Patients

The charts of all 122 patients who met the Vancouver criteria (definite or probable) [2] or ILAR criteria [1] for JPsA, and who were diagnosed and followed at the Rheumatology Clinic at The Hospital for Sick Children (SickKids), Toronto between 1985 and 2005 were reviewed.

### Data collection presentation and follow-up

Patients′ charts were reviewed for the following clinical variables at presentation and at each follow-up visit: height, weight, joint involvement, presence or absence of symmetric arthritis, nail pits, dactylitis, presence of psoriatic rash or psoriasis-like rash, and uveitis. All medications, including corticosteroid joint injections, were recorded for each visit. Patient visits at presentation 1, 3, 6, 12, 36, and 60 months following the first presentation and every 5 years thereafter were used**.** Serologic variables studied were: a) Rheumatoid Factor (RF) as detected by latex agglutination; a positive test result was defined as titer ≥ 2 on at least two occasions (data were available on 88% of patients); b) Anti-nuclear antibody (ANA) as detected by immunofluorescent microscopy using the Hep2 cell line. A positive result was defined as a titer ≥ 1:40 on at least one occasion (data were available on 88% of patients); and c) HLA B-27 antigen (data were available on 44.5% of patients).

The following complications were recorded at each visit: presence of a joint contracture, and presence of leg-length discrepancy. The Childhood Health Assessment Questionnaire (CHAQ) [14] was available at last follow-up in 73.1% of the patients. For patients with uveitis the final visual acuity and ocular complications were obtained [15].

### Definitions

The diagnosis of psoriasis was made by a rheumatologist and/or dermatologist. Rashes thought likely (but not definitively) to represent psoriasis were considered psoriasis-like.

Polyarticular involvement was defined by the involvement of ≥ 5 joints cumulatively at any point over the course of study. Oligoarticular arthritis was defined as involvement of < 5 joints: a)persistent oligoarthritis- < 5 joints throughout the disease course; or b)extended- ≥ 5 were involved at any point after the initial 6 months. Enthesitis was defined as tenderness at a tendinous, ligamentous, capsular, or fascial insertion into bone. Dactylitis was defined as digital swelling extending beyond the margins of the joints. Patients with dactylitis were not considered to have involvement of the corresponding joint(s) with arthritis unless it was specifically documented.

Symmetric arthritis was defined as being present if the number of affected joint pairs divided by the total number of joints involved was ≥50%. The following 11 joint pairs were used: shoulders, elbows, wrists, any metacarpophalangeal (MCP), any proximal interphalangeal (PIP) of the hand, hips, knees, ankles, any metatarsophlangeal (MTP), any PIP) of the foot, and the temporomandibular (TMJ) [16].

Inactive disease was defined as absence of clinically evident synovitis and enthesitis for a minimum of 3 months. Patients may have been receiving medication at the time of meeting the criteria for inactive disease.

Patients were excluded from inactive disease analysis if they were followed for < one year, or if insufficient data for the

Patients who were diagnosed with other rheumatic diseases such as systemic lupus erythematosus or had features of systemic JIA were excluded from the study.

### Definition of sub-types

Patients were divided into 4 groups depending on their clinical features: a) Oligoarticular course; b) RF negative (-) polyarticular course (divided into extended oligoarticular and polyarticular-onset; c) RF positive (+) polyarticular course; and d) enthesitis-related arthritis (ERA) (modified tILAR definition as psoriasis could be present) using ILAR criteria [1].

### Statistic methods

All descriptive data were expressed as the mean ± standard deviation (SD). Comparisons between groups were performed using Chi-Square Tests with Bonferroni corrections when applicable and ANOVA test with Tukey–Kramer Honestly Significant Difference correction for continuous variables (comparisons were for the following 4 groups: ERA, persistent oligoarticular, extended oligoarticular, and RF- polyarticular, and not polyarticular course). Kaplan-Meirer survival analysis was performed for time to first inactive disease, both on or off therapy, and was compared between the groups – the p value was calculated using Log Rank Chi-Square Test.

Comparisons of the continuous variables at presentation and at last follow-up were performed using paired t-test analysis.

Institutional Review Board approval was obtained for this study (no.0019980177).

## Results

The initial cohort consisted of 122 patients with JPsA: three were excluded due to insufficient follow-up data. The study cohort consisted of 119 patients – 109 patients had definite and 10 patients had probable JPsA by the Vancouver Criteria [2]. Ninety-nine of also fulfilled the ILAR criteria for JPsA [1]. The other 20 patients would have been excluded from the study if only ILAR criteria were used: a) 4 patients tested positive for rheumatoid factor on at least two occasions; and b) 16 patients also met the criteria for ERA. Ninety-nine patients had a definite psoriatic rash and 4 patients had a psoriatic-like rash. Thirty-three patients were diagnosed with psoriasis before the onset of arthritis (median 2.5 years mean of 3.4±3.1 years), 30 patients at the same time and in 40 patients the diagnosis of psoriasis was made after the onset of arthritis (median 2.9 mean of 4.2±4.0 years).

At the time of the first visit 93 patients fulfilled the Vancouver criteria with 87 patients having definite and 6 patients probable JPsA. Eighty-one of the 93 patients also fulfilled the ILAR criteria for JPsA at presentation (the other 12 met the criteria for more than one JIA sub-type). Ethnicity data was available for 56 patients (49%). The vast majority of patients (49/56) were of European ancestry (87.5%), one patient was African-Canadian, one Chinese-Canadian and 5 patients had a mixed ethnic origin.

### Demographic data

The mean age at presentation was 8.0±4.4 years with 80 females (67%) and 39 males (33%) (Table 1). The mean age at diagnosis was significantly lower for females with JPsA when compared to males (median 6.7 vs. 9.4 years) (mean 7.3±4.4 vs. 9.3±4.1 years) (p=0.03). The duration of follow-up ranged from 1 month to 16.8 years. There were no significant differences in the follow-up times among the groups (Table 1).

**Table 1:** Comparison of characteristics of patients with Juvenile Psoriatic Arthritis (JPsA) in the different groups

	**ERA N=16**	**Persistent oligoarticular N=44**	**Extended oligoarticular N=21**	**RF- polyarticular N=34**	**Polyarticular course N=55**	**Total cohort N=115**	**P value**
**Female:male**	1:2.2	2.7:1	1:1.6	1:3.9	2.5:1	2:1	0.006*
**Mean age at diagnosis in years**^**# **^**(median)**	11.6±2.2 (12.1)	7.7±4.3 (7.4)	6.6±5 (3.5)	7.4±4.2 (7.2)	7.1±4.5 (5.8)	8±4.4 (8.1)	0.003**
**Mean Length of follow-up in years**^**# **^**(median)**	4.8±2.3 (4.9)	5.9±3.8 (4.9)	7.2±0.9 (6)	6.6±0.7 (6.5)	6.8±4.6 (6.1)	6.2±4.1 (5.3)	0.3**
**Family history of psoriasis (percentage)**	8 (50)	26 (59)	12 (57)	22 (65)	34 (62)	68 (59)	0.8**
**Number of patients with positive ANA**^**a**^	1/14	14/40	11/19	13/29	24/49	39/103	0.07**
**Number of patients with positive HLA-B27**	6/15	3/18	0/8	1/8	1/17	10/50	0.0001*
**Number of patients with asymptomatic uveitis** **(acute uveitis)**	0 (5)	6 (0)	3 (0)	4 (0)	7 (0)	13 (5)	0.6*
**Number of patients with dactylitis**	3	11	11	11	22	36	0.09*

#- values are shown as mean plus/minus standard deviation.

a=Positive anti-nuclear antibody (ANA) defined as at least one positive ANA result.

* Anova.

**chi-square analysis.

The most common type of arthritis found was oligoarticular-onset in 65 patients (55%)- 44 patients (68%) had a persistent oligoarticular course and 21 (32%) had extended oligoarticular arthritis (polyarticular course). The median time to extension was 2.9 years (mean 3.1±2.3). Overall, 55 patients (46%) had polyarticular course arthritis (excluding patients with ERA). Features of ERA were present in 16 patients (14%): 7 patients had ≥ 5 joints during the disease course and 9 patients <5 joints. The least common course-type was RF+ polyarticular course, which was found in only 4 patients (3%) (not further analyzed because of sample size).

A positive family history of psoriasis was found in 68 patients: 40 had a first-degree relative with psoriasis and 28 had second-degree relative.

### Clinical findings

#### Joint involvement

##### a) Large joints

The most commonly involved large joints at both presentation and during the course of the disease were the knee (67%) and ankle (23% of patients) (Tables 2 and 3). Statistically significant differences in the frequency of large joint involvement at presentation were found for: hip (highest in ERA) (p=0.0012), ankle (highest in RF- polyarticular) (p=0.05) and wrist (lowest in ERA) (p=0.038) (Table 2). During follow-up, involvement of the hip (p=0.05), wrist (p=0.0004), and elbow (p=0.0002) differed among the groups (Table 3).

**Table 2:** Frequency of individual joint involvement at presentation (percentage)

	**ERA N=16**	**Persistent oligoarticular course N=44**	**Extended oligoarticular course N=21**	**RF-N=34**	**polyarticular course N=55**	**Total cohort N=115**	**P value**
**Knee**	6 (37.5)	32 (72.7)	15 (71)	24 (70)	39(70)	77 (66.9)	0.06
**Ankle**	5 (31.2)	9 (20.4)	7 (33)	17 (50)	24(44)	38 (33.0)	**0.05**
**Subtalar**	2 (12.5)	3 (6.8)	3 (14.3)	5 (14.7)	8(14.5)	13 (11.3)	0.7
**Hip**	5 (31.2)	5 (11.4)	0 (0)	0 (0)	0(0)	10 (8.7)	**0.0012**
**Shoulder**	1 (6.2)	1 (2.3)	1 (4.7)	3 (8.8)	4(7)	6 (5.2)	0.63
**Elbow**	1 (6.2)	3 (6.8)	3 (14.3)	9 (26.5)	12(21.8)	16 (13.9)	0.07
**Wrist**	1 (6.2)	4 (9.0)	7 (33)	13 (38.2)	20(36.3)	25 (21.7)	**0.038**
**MCP**	2 (12.5)	3 (6.8)	2 (9.5)	12 (35.3)	14(25.4)	19 (16.5)	**0.005**
**PIP of Hand**	3 (18.7)	6 (13.6)	2 (9.5)	18 (52.9)	20(36.3)	29 (25.2)	**0.0002**
**DIP of Hand**	2 (12.5)	2 (4.6)	0 (0)	6 (17.6)	6(10.9)	10 (8.7)	0.08
**TMJ**	1 (6.2)	2 (4.6)	6 (17.6)	6 (17.6)	6(10.9)	9 (7.8)	0.07
**Cervical spine**	0 (0)	2 (4.6)	0 (0)	0 (0)	1(1.8)	2 (1.7)	0.50
**Sacroiliac**	5 (31.2)	0 (0)	0 (0)	0 (0)	0(0)	5 (4.3)	**<0.0001**
Any small joint*	6 (37.5)	12 (27.3)	5 (24)	25 (73.5)	30(54.5)	48 (41.7)	**0.0001**

MTP metatarsal phalangeal joint.

PIP proximal interphalangeal joint.

MCP metacarpal phalangeal joint.

DIP distal interphalangeal joint.

*any small joint refers to: PIP of hands and feet, MCP, and MTP.

**Table 3:** Frequency of individual joint involvement during the course of the disease

	**ERA N=16**	**Persistent oligoarticular course N=44**	**Extended oligoarticular course N=21**	**RF- polyarticular N=34**	**Polyarticular course N=55**	**Total cohort N=115**	**P value**
Knee*	12 (75.0)	34 (77.2)	18 (85.7)	26 (76.4)	44(80)	90 (78.3)	0.8
**Ankle**	9 (56.0)	20 (45.0)	16 (76.2)	21 (62.0)	37(67)	66 (57.4)	0.1
**Subtalar**	4 (25.0)	4 (9.0)	9 (26.5)	7 (33.0)	16(29)	24 (20.1)	0.09
**Hip**	7 (43.7)	6 (13.6)	4 (19.0)	5 (14.7)	9(16.3)	22 (19.1)	**0.05**
**Shoulder**	2 (12.5)	2 (4.5)	3 (14.3)	8 (23.5)	11(0.2)	15(13.0)	0.1
**Elbow**	2 (12.5)	6 (13.6)	9 (43.0)	19 (56.0)	28(50.9)	36 (31.0)	**0.0002**
**Wrist**	3 (18.7)	13 (29.5)	13 (61.9)	23 (67.6)	36(65.4)	52 (45.2)	**0.0004**
**MCP**	3 (18.7)	5 (11.4)	9 (42.9)	21 (61.7)	30(54.5)	38 (33.0)	**<0.0001**
**PIP of Hand**	5 (31.0)	7 (15.9)	12 (42.8)	26 (76.5)	38(69)	51 (43.5)	**<0.0001**
**DIP of Hand**	3 (18.7)	3 (6.8)	5 (23.8)	10 (29.4)	15(27.2)	21 (18.3)	0.07
**TMJ**	1 (6.2)	5 (11.4)	7 (33.3)	10 (29.4)	17(30.9)	23 (20.0)	**0.04**
**Cervical spine**	0 (0)	4 (9.0)	3 (14.3)	5 (14.7)	8(14.5)	12 (10.3)	0.4
**Sacroiliac**	7 (43.7)	3 (6.8)	1 (5.0)	2 (6.0)	3(5.4)	13 (11.3)	**0.0002**
Any small joint **	7 (43.7)	12 (27.2)	3 (14.3)	4 (11.8)	48(87.3)	67 (58.0)	**<0.0001**

MTP metatarsal phalangeal joint.

PIP proximal interphalangeal joint.

MCP metacarpal phalangeal joint.

DIP distal interphalangeal joint.

* values are shown as number with percentage in brackets.

**any small joint refers to: PIP of hands and feet, MCP, and MTP.

##### b) Small joints

At presentation there was a statistically significant difference for any small joint involvement (p=.0005) and for MCP (p=0.005) and PIP of the hands (p=0.0002) (all highest in RF- polyarticular group) (Table 2). During the follow-up period again there was a statistically significant difference for any small joint (p<0.0001) and for PIP of the hands (p=0.0002) and MCPs (all highest in RF- polyarticular group) (p=0.005) (Table 3).

At presentation there was no significant difference for the frequency of DIP involvement but during follow-up DIP involvement differed (Table 3).

### TMJ

During the follow-up period we found that there was a statistically significant higher percentage of TMJ involvement in patients with RF- polyarticular and extended oligoarticular disease as compared to the ERA and persistent oligoarticular groups but at presentation (Tables 2 and 3).

### Sacroiliac (SI) joint

Five patients with ERA (31%) had SI joint involvement at presentation and 7 patients (44%) during the disease course. SI joint involvement was not seen in any of the other patients at presentation, and was only rarely seen during the follow–up in these patients (Tables 2 and 3).

### Symmetry of joint involvement

At presentation 45 patients of the total cohort (39%) had symmetric joint involvement and 52 patients (45%) during follow-up. There was an increased prevalence of symmetric arthritis for the RF- polyarticular and the extended oligoarticular groups compared to the persistent oligoarticular and ERA groups but not between the RF- polyarticular and extended oligoarticular groups (data not shown).

### Other features

There was no difference in the percentage of patients with dactylitis among the groups (p=0.14) (Table 1). At presentation patients with younger age of onset tended to have more dactylitis (R^2^=0.025; p=0.06).

Nail changes secondary to psoriasis was seen in 66 of the cohort (57%) with nail pitting seen in 64 patients and onycholysis in only 2 patients. Nail pitting was significantly associated with a higher incidence of DIP involvement at presentation but not during follow-up (p=0.0034 and p=0.6 respectively).

### Serology results

A positive ANA was associated with younger age (R^2^=0.07; p=0.0027) (Odds ratio 0.862 with confidence intervals of 0.78 -0.953).

There was a statistically significant difference for the presence of HLA-B27 among the groups (P<0.0001).

### Treatment

All patients were treated with NSAIDs at presentation and/or during follow-up. Methotrexate was the most commonly used second-line agent and was significantly more frequently used in patients with polyarticular course as compared persistent oligoarticular patients (p=0.01) but not to patients with ERA (Table 4). Within polyarticular course patients there was no difference in methotrexate use between RF- polyarticular and extended oligoarticular patients (data not shown). There was a significant difference in the use of other second-line agents when the polyarticular course group were compared to the persistent oligoarticular (45% vs. 23%, p= 0.02) but not when compared to the ERA group or between the 2 polyarticular course groups (data not shown) (Table 4). There were no differences in the use of oral prednisone and anti-tumour necrosis factor (TNF) therapy when comparing the polyarticular course group to the other groups, although patients with extended oligoarticular arthritis were significantly more likely to be treated with an anti-TNF agent than the other groups (24% vs. 6% p=0.05).

**Table 4:** Medication use during the course of the disease (percentage)

	**ERA N=16**	**Persistent oligoarticular course N=44**	**Extended oligoarticular course N=21**	**RF-polyarticular N=34**	**Polyarticular course N=55**	**Total cohort N=115**	**P value**
**Methotrexate**	4 (25.0)	8 (18.2)	10(47.6)	13(38.2)	23 (41.8)	35 (22.6)	**0.03**
**Anti-TNF agent**	2 (12.5)	1 (2.3)	6(28.5)	2(5.9)	7* (12.7)	10 (8.7)	0.16
**Etanercept**	1 (6.2)	1 (2.3)	5(23.8)	2(5.9)	7 (12.7)	9 (7.8)	0.15
**Infliximab**	1 (6.2)	0 (0)	1(4.7)	0(0)	1 (1.8)	2 (1.7)	0.26
**Any DMARD**	4 (25.0)	10 (22.7)	11(52.4)	14(41.2)	25 (45.4)	39 (33.9)	**0.04**
**Prednisone**	2 (12.5)	1 (2.3)	4(19)	3(8.8)	7 (12.7)	10 (8.7)	0.15

* one patient was treated with both etanercept and infliximab.

DMARD: Disease-Modifying Anti-Rheumatic Drug.

### Outcome measures

Twenty-one (18%) of the patients had a joint contracture during the course of their disease: small joint of the hand (10 patients), elbow in 5, and wrist and hip in 2 patients each. Seven patients had deviation of the jaw and one patient had restricted range of motion of the neck (Table 5). We found that patients with RF- polyarticular and extended oligoarticular course were more likely to have a contracture compared to the other groups at presentation but not during the disease course (Table 5). A leg-length discrepancy was found in only 3 patients (3%) at last follow-up with no significant difference among the groups.

**Table 5:** Comparison of outcome measures in the different groups (percentage)

	**ERA N=16**	**Persistent oligoarticular course N=44**	**Extended oligoarticular course N=21**	**RF-polyarticular N=34**	**Polyarticular course N=55**	**Total cohort N=115**	**P value**
**Contracture at any time**	0	5	8(38)	8(23.5)	16	21	**0.001**
**Contracture at last visit**	0	2	4(19)	8(23.5)	8	10	0.09
**Leg-length discrepancy**	2	9	2(9.5)	4(11.7)	6	17	0.4
Time to last CHAQ*^# ^**from diagnosis in years**	4.5±3.2	5.7±3.8	7.4±5.3	7.2±4.4	7.3±4.7	6.3±4.3	not significant
**Value of last CHAQ**	0.25± 0.40	0.23±0.40	0.23±0.34	0.32±0.36	0.28±0.40	0.26±0.4	not significant

* CHAQ- Childhood Health Assessment Questionnaire.

#- values are shown as mean plus/minus standard deviation.

A CHAQ score was available in 86 patients (75%) at the last visit after a mean time of arthritis of 6.3±4.3 years. The mean CHAQ scores were similar among the groups (Table 4).

Assessment of inactive disease was available for 109 patients. All but 7 patients (4 with polyarticular, 2 with oligoarticular and 1 with ERA) had inactive disease at least once during the follow-up period. At the last follow-up 63 patients (55%) had inactive disease; there were no differences among the groups for percentage of patients with inactive disease on medication. At the last follow-up 33 patients (30%) were inactive off all medication and again there was no difference among the groups.

**Figure 1:**
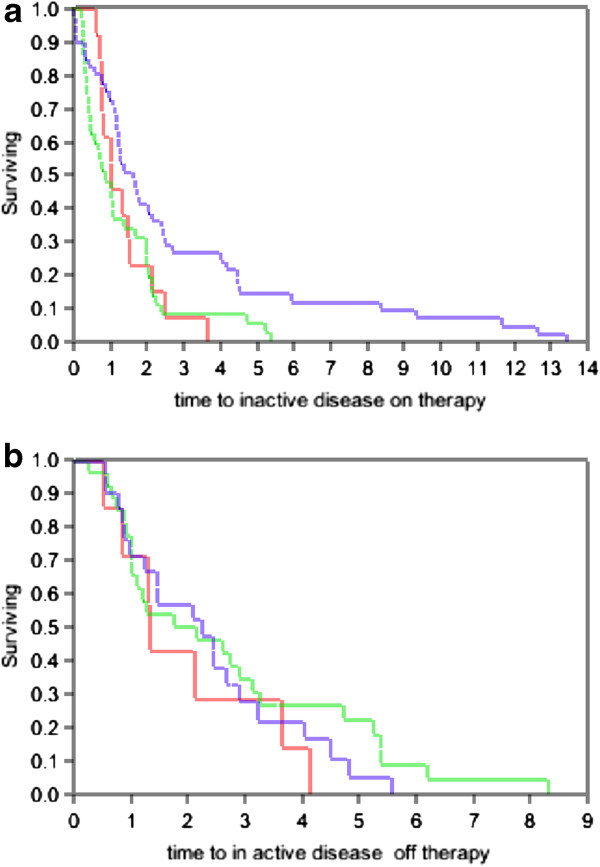
**a and b show Kaplan-Meir survival analysis for the time to first inactive disease for patients on (Figure** 1**a) and off (Figure** 1**b**) **therapy. a**: Patients on therapy, Kaplan-Meir survival analysis for the time to first inactive for patients with persistent oligoarticular arthritis (green line), ERA group (red line) and polyarticular course arthritis (blue line) for patients on therapy. The x-axis is time in years and the y-axis shows the percentage of patients with active arthritis. **b**: Patients off therapy. Kaplan-Meir survival analysis for the time to first inactive for patients with persistent oligoarticular arthritis (green line), ERA group (red line) and polyarticular course arthritis (blue line) for patients off therapy. The x-axis is time in years and the y-axis shows the percentage of patients with active arthritis.

Using Kaplan-Meir survival analysis, the median time to inactive disease on therapy for the total cohort was 1.23 years (mean 2.1 ±0.3 years) and the median time to inactive disease off therapy was1.8 years (mean 2.5±0.26 years) (Figure 1). There was no significant difference among the groups in mean time to first inactive disease on therapy (p=0.4) or off therapy (p=0.2) (Figure 1).

### Growth

Height and weight data were available at the first and last visit in 53 patients (46%). The median percentile for weight at last follow-up at 68.5 (mean 65.2±26.4) was significantly higher when compared to the first visit that was 57.6 (mean 54.2±31.3) (p=0.0014) with no significant differences among the groups.

There was no significant change in the height percentiles from the first visit to the last visit (medians 54.9 vs. 61.8) (means 55.7±29.4 vs. 53.6±29.6) (p=0.77).

### Uveitis

Chronic uveitis occurred in 13 patients (11%) of the total cohort. There was no significant difference among the groups (p=0.63). Acute uveitis developed in 5 patients and all 5 patients had ERA.

## Discussion

It is recognized that patients with JPsA may be a heterogeneous group of patients who may present with features of similar to the other JIA subtypes. Two different classification systems of JPsA have been proposed, with the ILAR classification gaining increasing usage [1,2]. Our findings are consistent with previous studies that suggested patients with JPsA comprise distinct populations that can be differentiated by the age of onset and clinical features [6,11,17,18]. These results are similar to studies of adult patients with PsA that demonstrated subsets based on number of and/or location of joint involvement, symmetry and features of spondylitis [19-23].

The diagnosis of JPsA frequently occurs in patients who had been previously diagnosed with other forms of JIA with development of the diagnostic rash month to years later. We found that 1/3 of patients were diagnosed with another JIA sub-type prior to the diagnosis of JPsA after a mean of 4.2 years. In most of these patients a diagnosis of JPsA was not suspected based on clinic findings although some had a family history of psoriasis. In addition 3 patients with systemic JIA and psoriasis (data not shown), and 4 with RF+ polyarticular JIA and psoriasis could not be clinically differentiated from others with systemic or RF+ polyarticular JIA.

The most common course of arthritis found in the cohort was polyarticular course, present in 52% of patients which is similar to our incidence of polyarticular-course JIA in patients without psoriatic arthritis [24] but slightly lower than previous studies of JPsA [5,6,10]. Patients with polyarticular course could be distinguished from the other subtypes as they had a worse outcome, more severe clinical course, had more contractures, and longest mean time to first inactive disease. The pattern of arthritis differed as they were most likely to have involvement of the small joints of the hands and feet, to have symmetric disease, to require methotrexate and least likely to have hip and SI joint involvement. These findings would suggest that patients with polyarticular course JPsA form a distinct subset of JPsA patients that closely resemble patients with polyarticular course JIA without psoriasis.

The most common onset type was oligoarticular arthritis, present in 55% of cases with extension in approximately 1/3 of cases; an extension rate that is similar to that seen non-psoriatic oligoarticular onset patients [24-27]. Similarly, the clinical course of patients with both persistent oligoarthritis and extended oligoarthritis more closely resembled JIA clinical sub-types without psoriasis rather than other JPsA as a whole. At presentation found the frequency of wrist involvement of olgioarticular patients was closer to the frequency reported in oligoarticular JIA patients than the reported frequency of JPsA patients not subdivided [16,28,29]. These findings suggest that the clinical course of patients with oligoarticular onset JPsA more closely resembles that of oligoarticular JIA rather than an unsubseted JPsA cohort.

A previous study had suggested that there were 2 distinct groups of patients with JPsA based on age of onset of disease with the older patients resembling ERA patients without psoriasis [11]. These older patients generally resembled the patients in our study with ERA and psoriasis in the pattern of joint involvement. Acute uveitis was only found in this group. Taken together these findings suggest that these patients older onset male patients more resemble patients with ERA without psoriasis than patients with other courses of JPsA. The prevalence of HLA-B27 was similar to previous reports in both ERA and JPsA with ERA features [5,27,30]. We suggest that this subgroup of patients should be classified and treated in the same manner as patients with ERA without psoriasis rather the ILAR current classification of undifferentiated. However, long-term studies are required to confirm this.

The overall outcome of patients in our study was excellent as during the course of disease 88% achieved inactive disease on therapy and 50% inactive disease off therapy. These rates are similar to previously reported remission rates depending on the definition of remission and the duration of the follow-up period [31]. The majority of our patients did not have arthritis at last follow-up, 30% of patients were without active arthritis off all medication and only 10% had a contracture. We found that our patients tended to have linear growth along the predicted percentiles but that weight percentiles significantly increased. The significance of this finding is unknown but there have been reports that adults with psoriasis tend to be obese [32].

## Conclusions

In conclusion we suggest that JPsA may comprise 4 distinct groups that are similar to non-JPsA JIA regarding presentation, disease course, uveitis associations, response to treatment and outcome. Future studies are required to determine if our results are seen in other cohorts. If other large independent cohorts confirm our findings then we suggest that the presence of psoriasis may have little clinical relevance in the outcome and response to therapy of children with JIA and therefore we may consider psoriasis as an extra-articular manifestation seen in JIA, similar to uveitis, rather a feature requiring a distinct classification grouping.

## Competing interest

The authors declare that they have no competing interests.

## Authors’ contribution

YAB was involved in all aspects of the study. PT was involved in the statistical analysis of the study and in preparation and review of the manuscript. RS was involved in patient recruitment and review of the manuscript. SD was involved in acquisition of the data. BF was involved in patient recruitment and review of the manuscript. RL was involved in patient recruitment and review of the manuscript. ST was involved in acquisition of the data and review of the manuscript. SMLT was involved in patient recruitment and review of the manuscript. EDS was involved in all aspects of the study. All authors have read and approved the final manuscript.
